# Spinal Anesthesia Increases the Frequency of Extubation in the Operating Room and Decreases the Time of Mechanical Ventilation after Cardiac Surgery

**DOI:** 10.21470/1678-9741-2019-0433

**Published:** 2021

**Authors:** Gustavo Siqueira Elmiro, Artur Henrique de Souza, Stanlley de Oliveira Loyola, Maurício Lopes Prudente, Celina Lumi Kushida, José Onofre de Carvalho Sobrinho, Fabiano Zumpano, Giulliano Gardenghi

**Affiliations:** 1 Department of Anesthesiology Service, ENCORE Hospital, Aparecida de Goiânia, Goiás, Brazil.; 2 Department of Cardiovascular Surgery, ENCORE Hospital, Aparecida de Goiânia, Goiás, Brazil.; 3 Department of Intensive Care Unit, ENCORE Hospital, Aparecida de Goiânia, Goiás, Brazil.; 4 Department of Hemodynamic Service, ENCORE Hospital, Aparecida de Goiânia, Goiás, Brazil.; 5 Department of Scientific Coordination, ENCORE Hospital, Aparecida de Goiânia, Goiás, Brazil.

**Keywords:** Anesthesia, Spinal, Airway Extubation, Respiration, Artificial, Cardiac Surgical Procedures, Clinical Protocols, Operation Rooms, Postoperative Period

## Abstract

**Introduction:**

The delayed extubation of patients undergoing mechanical ventilation (MV) in the postoperative period of cardiac surgery (CS) is associated with mortality. The adoption of spinal anesthesia (SA) combined with general anesthesia in CS influences the orotracheal intubation time (OIT). This study aims to verify if the adoption of SA reduces the time of MV after CS, compared to general anesthesia (GA) alone.

**Methods:**

Two hundred and seventeen CS patients were divided into two groups. The GA group included 108 patients (age: 56±1 years, 66 males) and the SA group included 109 patients (age: 60±13 years, 55 males). Patients were weaned from MV and, after clinical evaluation, extubated.

**Results:**

In the SA group, considering a 13-month period, 24% of the patients were extubated in the operating room (OR), compared to 10% in the GA group (*P*=0.00). The OIT was lower in the SA group than in the GA group (SA: 4.4±5.9 hours *vs*. GA: 6.0±5.6 hours, *P*=0.04). In July/2017, where all surgeries were performed in the GA regimen, only 7.1% of the patients were extubated in the OR. In July/2018, 94% of the surgeries were performed under SA, and 64.7% of the patients were extubated in the OR (*P*=0.00). The OIT on arrival at the intensive care unit to extubation, comparing July/2017 to July/2018, was 5.3±5.3 hours in the GA group *vs*. 1.7±3.9 hours in the SA group (*P*=0.04).

**Conclusion:**

The adoption of SA in CS increased the frequency of extubations in the OR and decreased OIT and MV time.

**Table:** 

Abbreviations, acronyms & symbols				
ASD	= Atrial septal defects		MAC	= Monitored anesthesia care
BIS	= Bispectral Index Value		MV	= Mechanical ventilation
CABG	= Coronary arterial bypass grafting		MVS	= Mitral valve surgery
CPB	= Cardiopulmonary bypass		OIT	= Orotracheal intubation time
CS	= Cardiac surgery		OR	= Operating room
ET	= End-tidal		SA	= Spinal anesthesia
EV	= Endovenous		SAVR	= Surgery of aortic valve replacement
GA	= General anesthesia		SR	= Suppression rate
ICU	= Intensive care unit		TOF	= Train-of-four
LVEF	= Left ventricular ejection fraction			

## INTRODUCTION

Cardiac surgery (CS) encompasses a series of highly complex procedures aimed at treating diseases of high morbidity and mortality of the circulatory system, the most common being cardiac valve replacement, myocardial revascularization, correction of aortic aneurysms/dissections, and communications between the left and right sides heart chambers. In accordance with the above, patients presenting with such diseases are generally known to be more serious cases than the surgical population in general, either due to the disease causing the surgery or to other comorbidities that affect the circulatory system. Despite the improvements in techniques applied in CS and the development of protection strategies, damage may eventually be seen in the postoperative period. It is known that thoracic epidural anesthetic techniques can reduce risk of perioperative myocardial infarction and decrease the response to perioperative stress. It is also known that postoperative analgesia is often superior, which can reduce systemic opioid consumption, orotracheal intubation time (OIT), and pulmonary morbidity^[1]^. The choice of an adequate anesthetic technique and the conduction of the organic changes intrinsic to these procedures are of major importance for the survival and quality of life of the individuals who undergo this type of treatment. Anesthesia for such procedures should invariably include general anesthesia (GA), because of the need for long-term anesthesia, gas exchange control, and potential risk of cardiac arrest and death in surgery. Neuraxial analgesia in the context of CS is performed using low concentrations of local anesthetics or opioids, resulting in a partial sensory blockade. It is important to highlight that Zangrillo et al.^[1]^ demonstrated that spinal analgesia with opioids alone may not offer significant clinical benefits when compared to GA.

In the anesthesiological practice, spinal anesthesia (SA) is a safe technique, when well indicated, and is also capable of bringing a series of benefits to patients, such as minor postoperative pain, minor surgical bleeding, reduction of sympathetic activity with improvement of blood flow to the various organs, protection against β-receptor down regulation, reduction of the endocrine-metabolic response to surgical stress (especially in large surgeries), reduction of the risk of venous thromboembolism, and earlier return of intestinal activity. In addition, it consists of profound sensory analgesia (by the abolition of all sensory stimuli transmissions), as well as motor and sympathetic blockade in the anesthetized area, obtained from a pharmacologic denervation at the level of the spinal cord by using highly-concentrated local anesthetics, usually achieved by the spinal approach^[2]^. In CS, satisfactory neuraxial anesthesia is achieved with a sensory block from T-1 downward and total body sympathectomy^[2]^. This technique may drastically reduce the use of analgesic drugs in the postoperative period which may result in shorter mechanical ventilation (MV) and associated complications, known to contribute to increased mortality and hospital stay.

It is important to mention that, by definition, anesthesia would be the abolition of the transmission of any afferent impulse (which necessarily requires high concentrations of local anesthetics because there must be blocking of both thin and small myelinated fibers, as well as thick and richly myelinated fibers), whereas analgesia consists of only reducing the transmission of nociceptive stimuli, which are carried by thinner fibers with little or no myelination and is easily achieved with opioids or low concentrations of local anesthetics.

This study aims to evaluate the repercussions of the inclusion of SA to GA in the postoperative evolution of patients submitted to CS with extracorporeal circulation, verifying if the adoption of SA can reduce the time of MV after CS, compared to GA alone.

## METHODS

This is an analytical and retrospective study in which patients were evaluated through the analysis of medical records of the ENCORE Hospital, in Aparecida de Goiânia (Goiás, Brazil). All the information was extracted from the database for statistical analysis in a confidential manner to maintain the integrity and privacy of the patients. The research was submitted to the Ethics Committee indicated by the Brazil Platform, under CAAE number 10217013.20000.0030. All the procedures followed the committee's ethical standards research in humans and the 1975 Helsinki Declaration, revised in 2000.

### Inclusion Criteria

In this study, all patients undergoing CS with extracorporeal circulation at the service, considering the period from July 2017 to July 2018, were included in the analysis. The surgeries were coronary arterial bypass grafting (CABG), mitral valve surgery (MVS), surgery of aortic valve replacement (SAVR), atrial septal defects, aortoplasty, or combined procedures as CABG + MVS and CABG + SAVR. Only adult patients (≥ 18 years old) were enrolled.

### Exclusion Criteria

All individuals with incomplete data in medical records were excluded.

### Study Protocol

#### Spinal Anesthesia Protocol

Patients were premedicated with oral midazolam (7.5 mg) 30 minutes preoperatively. On arrival at the operating room (OR), they were monitored with cardioscopy, oximetry, and neuromuscular blockade (train-of-four [TOF]), they were offered oxygen on nasal catheter, a peripheral vein was catheterized with a 16 or 14-gauge endovenous (EV) device, and the radial artery was catheterized with a 20 or 18-gauge EV device, both after local skin anesthesia with lidocaine. After venoclysis, surgical antibiotic prophylaxis was administered - epsilon aminocaproic acid (50 mg/kg), dexamethasone (10 mg), vitamin C (1 g), and ranitidine (50 mg), followed by continuous infusion of epsilon aminocaproic acid (25 mg/kg/h), interrupted only at the end of the procedure.

For the intrathecal injection, patients were placed in the sitting position, and the skin was prepared and draped in an aseptic fashion. Patients received the intrathecal injection; placement of the spinal bupivacaine was accomplished in the region of the fourth to fifth lumbar median interspace using a 26-gauge spinal needle. A maximum of three attempts were made to successfully locate the subarachnoid space. Similarly, in the event of blood returning in the spinal needle, the spinal anesthetic technique was discontinued and heparinization was delayed for one hour. When clear, free-flow of cerebrospinal fluid was established, 0.5% preservative-free, hyperbaric bupivacaine (6-8 ml) with sufentanil (20-25 mcg) and morphine (3 mcg/kg) were injected quickly. All patients were placed in 30° Trendelenburg position for seven minutes. The level of SA was determined by checking for the onset of loss of cold sensation in the T1 level of anesthesia. The anesthesiologists of our group developed the present protocol for SA based on the protocols proposed by three groups of researchers^[2-4]^. The local anesthetic dosage in our protocol was based on two studies^[2,4]^, which combined the use of hyperbaric bupivacaine and local anesthetics as sufentanil and/or morphine.

Next, preoxygenation was performed with a 100% O_2_ bag-valve-mask system for 2-3 minutes and anesthesia was subsequently induced with propofol (1-1.5 mg/kg), cisatracurium (0.15 mg/kg), and ketamin-S (0.5-1 mg/kg) guided by Bispectral Index Value (BIS). Sevoflurane (1-2%, expired) was titrated to maintain BIS of 40-60 and suppression rate (SR) of 0. After TOF = 0, periglottic anesthesia with 2% lidocaine (5 ml) + 1% ropivacaine (5 ml) was performed with a mucosal atomizer and orotracheal intubation, followed by confirmation with capnography, auscultation, and tube fixation with a tape. Subsequently, the central venous access puncture was performed by the surgeon. During cardiopulmonary bypass (CPB), hypnosis was maintained with sevoflurane administered through the perfusion machine and in accordance with the BIS and gas analyzer installed on the machine's gas outlet for a BIS 40-60, SR of 0, and end-tidal (ET) sevoflurane at 0.7-1 monitored anesthesia care (MAC).

Heparin was administered to establish an activated clotting time > 480 s. CPB circuit was primed with a small crystalloid volume (1 l). CPB was conducted with a roller pump and a membrane oxygenator at 33-35°C. Flow was maintained at 2.2-2.5 l min^-1^ m^-2^. Alpha-stat blood gas management was used throughout the procedure. Epsilon aminocaproic acid was continuously given with infusion pump. During CPB, all patients received ET sevoflurane 0.7-1 MAC, titrated to maintain a BIS of 40-60 and SR of 0. Lactated Ringer was used as required for volume resuscitation in the post-CPB period. Protamine (1 mg/100 U of heparin) was given for heparin reversal. Blood product use was at the discretion of the attending anesthesiologist. Fluid balance for the intraoperative period was calculated by subtracting the urine output as well as weighed and suctioned blood loss from the total fluids administered and the CPB balance. Intravenous nitroprusside and metaraminol were also used to control hemodynamic parameters, when necessary. Muscle relaxation was reversed with the administration of intravenous neostigmine (0.04 mg/kg) and atropine (0.02 mg/kg), if necessary. MV was set to a tidal volume of 6 ml/kg of the predicted body weight, with positive end-expiratory pressure of 8 cmH_2_O, and respiratory rate was titrated to maintain an arterial carbon dioxide partial pressure of 35-40 mmHg. The oxygen inspired fraction was titrated aiming for an oxyhemoglobin saturation > 92%.

After subcutaneous plane closure, dipyrone (2 g) and ondansetron (8 mg) were administered. After that, administration of inhaled anesthetics was interrupted to awake the patient at the end of surgery, if there were criteria for extubation in OR. If the patients presented BIS > 85, obeyed commands, presented tidal volume > 6 ml/Kg with pressure support of 8 cmH_2_O, and lack of bleeding or high vasoactive drug levels, then they were extubated in the OR. If there were no conditions for extubation in the room, propofol (50 mg) was administered before the transport to the intensive care unit (ICU) if the patient started awakening.

#### General Anesthesia Protocol

Patients were premedicated with oral midazolam (7.5 mg) 30 minutes preoperatively. On arrival at the OR, they were monitored with cardioscopy, oximetry, and TOF, they were offered oxygen on nasal catheter, a peripheral vein was catheterized with a 16 or 14-gauge EV device, and a radial artery was catheterized with a 20 or 18-gauge EV device, both after local skin anesthesia with lidocaine. After venoclysis, surgical antibiotic prophylaxis was administered, epsilon aminocaproic acid (50 mg/kg), dexamethasone (10 mg), vitamin C (1 g), and ranitidine (50 mg), followed by continuous infusion of epsilon aminocaproic acid (25 mg/kg/h), interrupted only at the end of the procedure. Next, preoxygenation was performed with a 100% O_2_ bag-valve-mask system for 2-3 minutes and general anesthetic venous induction with sufentanil (0.3 mcg/kg) + propofol (1-1.5 mg/kg) + ketamin-S (0.5-1 mg/kg) + cisatracurium (0.15 mg/kg) guided by BIS to maintain values of 40-60 with SR of 0. After TOF = 0, periglottic anesthesia with 2% lidocaine (5 ml) + 1% ropivacaine (5 ml) was performed with a mucosal atomizer and orotracheal intubation, followed by confirmation with capnography, auscultation, and tube fixation with tape.

Subsequently, the central venous access puncture was performed by the surgeon, and GA was maintained with sevoflurane, guided by BIS and gas analyzer. Supplementary doses of sufentanil were administered during sternotomy and CPB entry according to hemodynamic responses. During CPB, hypnosis was maintained with sevoflurane administered through the perfusion machine and in accordance with BIS and gas analyzer installed on the machine's gas outlet for a BIS 40-60, SR of 0, and ET sevoflurane 0.7-1 MAC. After subcutaneous plane closure, surgical wound infiltration was performed by the surgeon with NovaBupi 0.5% with vasoconstrictor, dipyrone (2 g) and ondansetron (8 mg) were also administered. After that, administration of inhaled anesthetics was interrupted to awaken the patient at the end of surgery, if there were criteria for extubation in OR. If there were no conditions for extubation in the room, propofol (50 mg) was administered before the transport to the ICU if the patient started awakening.

#### MV Weaning Protocol in Intensive Care Unit

After surgery, patients admitted to the ICU were weaned from MV in pressure support mode (pressure offered aiming to maintain a tidal volume of 6 ml/kg of the predicted body weight). The oxygen inspired fraction was titrated aiming for an oxyhemoglobin saturation > 92%. The positive end-expiratory pressure was 8 cmH_2_O. If the patient responded to verbal commands with an effective cough and without signs of ventilatory discomfort, he/she was extubated. The OIT was considered from the arrival at the ICU.

#### Outcomes

The analyzed outcomes in this study were the frequency of extubation in the OR, time for extubation of patients admitted to ICU, length of stay at the hospital, reintubation rate, and in-hospital mortality.

### Statistical Analyses

Categorical data were expressed in absolute (n) and relative (%) frequency. Semicontinuous and continuous variables were expressed as a mean and standard deviation. Variables were tested for normality distribution by applying the Shapiro-Wilk test. Patients' demographic, intraoperative, and postoperative characteristics data were examined using unpaired t-tests and chi-square analyses. The nonparametric measurements were tested by Fisher's test. A *P*-value < 0.05 was considered statistically significant for all tests. Statistical analyses were performed using Statistica 10.0 Software (Statsoft Inc., Tulsa, Oklahoma, United States of America).

## RESULTS

Retrospectively, through the analysis of medical records, 217 patients were divided in the GA group (108 patients) and the SA group (109 patients). There were no significant differences in the preoperative patient characteristics (Table 1).

**Table 1:** Patients' anthropometrics and clinical data.

	Spinal anesthesia (n=109 patients)	General anesthesia (n=108 patients)	*P*-values
Age (years)	60.3±13.6	56.7±15.1	0.06
Male sex, n (%)	60 (55.0%)	66 (61.1%)	0.24
Smoking, n (%)	36 (33.1%)	29 (27.0%)	0.43
Hypertension, n (%)	62 (57.4%)	70 (52.2%)	0,3
Dyslipidemia, n (%)	58 (53.6%)	52 (48.1%)	0.43
Diabetes, n (%)	24 (21.9%)	18 (18.9%)	0.17
LVEF (Teicholz), n (%)			0.56
≥ 50%	61 (56.0%)	56 (51.6%)	
40-49%	33 (30.0%)	36 (33.0%)	
< 40%	15 (14.0%)	17 (15.4%)	
Type of surgery			0.68
CABG	62 (56.9%)	51 (47.2%)	
MVS	22 (20.2%)	20 (18.5%)	
SAVR	12 (11.0%)	24 (22.2%)	
ASD	2 (1.8%)	4 (3.7%)	
Aortoplasty	9 (8.3%)	3 (2.8%)	
CABG + MVS	-	3 (2.8%)	
CABG + SAVR	2 (1.8%)	3 (2.8%)	

Data presented in absolute number and percentage (%) ASD=atrial septal defects; CABG=coronary arterial bypass grafting; LVEF=left ventricular ejection fraction; MVS=mitral valve surgery; SAVR=surgery of aortic valve replacement

Intraoperative and postoperative patient characteristics are shown in Table 2.

**Table 2:** Comparisons of groups in the intraoperative and postoperative periods.

	Spinal anesthesia (n=109 patients)	General anesthesia (n=108 patients)	*P*-values
Time of extracorporeal circulation, minutes	78.1±30.5	70.5±27.8	0.06
Drains, n (%)			0.74
Mediastinal	59 (53.8%)	56 (52.1%)	
Pleural	4 (3.8%)	7 (6.3%)	
Pleural + mediastinal	46 (42.3%)	45 (41.7%)	
Frequency of extubation in the OR, n (%)	26/109 (23.9%)	11/108 (10.2%)	0.00
Time for extubation of patients admitted to ICU, minutes	269.8±331.7	367.9±341.5	0.03
Length of stay at ICU, days	4.9±2.5	5.4±3.2	0.22
Length of stay at ward, days	4.3±3.6	4.1±2.1	0.23
Reintubation rate, n (%)	2/109 (1.8%)	2/108 (1.8%)	0.44
Mortality, n (%)			0.89
ICU	8/109 (7.3%)	7/108 (6.5%)	
Ward	2/101 (1.9%)	3/101 (2.9%)	
Total	10/109 (9.1%)	10/108 (9.2%)	

Data presented in absolute number and percentage (%) or as mean ± standard deviation ICU=intensive care unit; OR=operating room

**Fig. 1:**
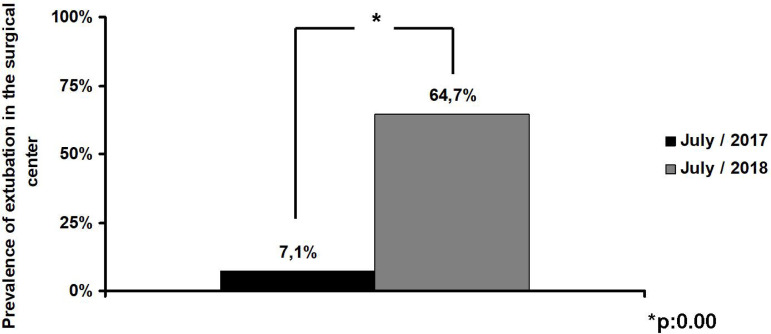
Prevalence of extubation at the surgical center, comparing July/2017, when all surgeries were performed under general anesthesia, against July/2018, when 94% of all surgeries were performed under spinal anesthesia.

In July/2017, when all surgeries were performed in the GA regimen, only 7.1% of the patients were extubated in the OR. In July/2018, 94% of the surgeries were performed under SA, and 64.7% of the patients were extubated in the OR (Figure 1).

The complication rate in our study related to spinal puncture for SA application was nonexistent. No neurological events or hematoma at the puncture site were observed in this retrospective series.

## DISCUSSION

Our study was able to show a decrease in OIT in patients admitted to the ICU, as well as a higher frequency of extubation in the OR in the evaluated sample. Studying and discussing ways of performing anesthesia in CS is important, since the applied surgical techniques have evolved, such as minimally invasive surgeries and new valvuloplasty techniques. Anesthesiology needs to deal with situations such as facing longer surgical times, seeking, as a specialty, to decrease the postoperative intubation time and to early restore the level of consciousness, which invariably results in less postoperative complications, such as risk of aspiration, incidence of pneumonia, or other opportunistic infections related to the length of hospital stay. SA may offer potential benefits in CS. It has been described that inflammatory responses have been correlated with a variety of adverse outcomes (cardiovascular, pulmonary, renal, hematologic, and neurologic dysfunction). In CS, SA as a supplement to GA can attenuate these responses, considering that it favorably alters the net inflammatory response based on measurements of serum biomarkers^[4]^. Decreases in serum concentrations of epinephrine, norepinephrine, and cortisol in the post-CPB period for CABG surgery were also observed^[3]^.

In order to seek new anesthetic techniques in CS, several authors have been looking for options. Chaney mentioned that intrathecal and epidural techniques potentially promote intense postoperative analgesia and stress response attenuation after CS. The author emphasize that such techniques are not risk-free and should be further investigated, in order to reduce morbidity and mortality^[5]^. Bignami et al.^[6]^ were able to demonstrate in an elegant meta-analysis that epidural analgesia on top of GA reduced the time on MV, agreeing with our study. This meta-analysis also demonstrated important reductions in the incidence of perioperative acute renal failure and in the composite endpoint of mortality and myocardial infarction in patients undergoing CS. Zhang et al.^[7]^, in another meta-analysis, analyzed the repercussions of thoracic epidural anesthesia in outcomes like hospital length of stay and time to tracheal extubation in patients undergoing CS. It was found that the epidural anesthesia could reduce the risk of complications such as supraventricular arrhythmias, in-hospital or ICU lengths of stay, and time to tracheal extubation. Specifically considering SA, Barbosa et al.^[8]^ mention that the adoption of SA was unable to decrease mortality and in-hospital length of stay. This agrees with our study, which was also unable to relate the protective effect of SA in these two outcomes.

Nashef et al. suggest that in-hospital mortality in CS is around 4%^[9]^. In our study, in-hospital mortality was around 9.1%. Emergency surgeries as well as surgeries of very serious patients, coming from needy regions of the metropolitan region of Goiânia, which often managed late referral from the local public health system to perform the surgical procedure in our hospital, may justify this significant increase in mortality. Lisboa et al., also addressing mortality in CS, showed that the mortality rates in their study, performed in a large Brazilian center, revolved around 8.5% in valve surgeries. The average global mortality was 7.0%, being 4.9% among the elective procedures^[10]^.

It is well known that CS is a type of elective surgery where immediate extubation is not considered a routine procedure. Hemmerling^[11]^ attributes to the team of surgeons and anesthesiologists the ability to perform early extubation or not. He highlights the fact that if working with surgeons where the outcome is usually sub-standard, the surgery takes a very long time, and postoperative complications are frequent, installation of an immediate extubation program after CS might be fruitless and inherent to dangers to morbidity and mortality of patients. If good surgeons with good outcomes and low rates of complications are acting, it provides the best environment for an immediate extubation program after CS. The opinion of the authors of the present study is that anesthesiology teams must consider the option of SA in CS, which could optimize tracheal tube removal and avoid complications related to long unconsciousness, as delirium, for example. Such complications are very common in the postoperative period of CS, especially when high doses of intravenous opioids are administered, generally prolonging intubation times, which should be avoided in the standard care of patients after CS^[3]^. It is expected that, in a close future, anesthesiologists will have to direct their patient care not only toward hemodynamic stabilization but also to the goal of immediate extubation. Choosing anesthetic techniques which allow immediate extubation is mandatory for that. Lower dose opioid strategies and combination with regional techniques can be an option, monitoring of anesthetic depth (*e.g*., with BIS) is also a useful tool to allow immediate extubation. Hemmerling also cites that the maintenance of body temperature is a key factor of immediate extubation strategies^[11]^.

Another important aspect is related to the risk of spinal cord injury from hematoma, which remains somewhat unclear. Evidence is still limited in CS patients. In the study conducted by Lee et al., no patient had complaints regarding the occurrence of dyspnea and upper and lower extremity paresthesia and weakness as a result of the intrathecal injection^[3]^. This is in agreement with our study, where no complications related to SA were observed.

One may consider that hypotension is another potential deterrent for anesthesiologists considering neuraxial techniques in CS. Casalino et al. described the risk factors for arterial hypotension associated with high thoracic epidural anesthesia in CS. They found an incidence of 17.6% for hypotension during the 30-minute interval after the administration of bupivacaine and alfentanil into the epidural space, easily corrected by administering a low dose of vasoconstrictors (continuous infusion of noradrenaline at an initial dose of 0.01 µg/kg/min), without untoward effects or complications. These hypotensive events were correlated with female sex^[12]^. Interestingly, a study previously cited in this article reported no difference between the intrathecal group and the group treated by GA in the need for phenylephrine or volemic resuscitation in the postoperative period of CS^[3]^. In agreement with the last sentence, our data collection did not show hemodynamic instability events associated with the adoption of SA.

### Limitations

This study has some drawbacks that need to be considered. In the opinion of the authors, the major limitation is related to the fact that the present study is a retrospective one, which may influence the results reported here. Another important point is related to the diversity of surgeries included in the analysis, which included emergency surgeries along with elective surgeries, which may have directly affected the observed mortality rates. Patients undergoing heart valve surgery may have a different postoperative course than those undergoing CABG.

## CONCLUSION

In conclusion, our study provides evidence that the adoption of SA in CS increased the frequency of extubations in the OR and decreased the OIT and MV time. No differences were observed in length of stay, reintubation rates, or mortality in the analyzed patients.

**Table:** 

Authors' roles & responsibilities	
GSE	Substantial contributions to the conception of the work; and the acquisition and analysis of data for the work; drafting the work and revising it; final approval of the version to be published
AHS	Substantial contributions to the conception of the work; and the acquisition and analysis of data for the work; revising the work; final approval of the version to be published
SOL	Substantial contributions to the conception of the work; and the acquisition and analysis of data for the work; drafting the work and revising it; final approval of the version to be published
MLP	Substantial contributions to the analysis of data for the work; revising the work; final approval of the version to be published
CLK	Substantial contributions to the acquisition and analysis of data for the work; final approval of the version to be published
JOCS	Substantial contributions to the acquisition and analysis of data for
FZ	Substantial contributions to the analysis of data for the work; revising the work; final approval of the version to be published
GG	Substantial contributions to the analysis and interpretation of data for the work; drafting the work and revising it; final approval of the version to be published
